# IL-25 Could Be Involved in the Development of Allergic Rhinitis Sensitized to House Dust Mite

**DOI:** 10.1155/2017/3908049

**Published:** 2017-08-23

**Authors:** Dae Woo Kim, Dong-Kyu Kim, Kyoung Mi Eun, Jun-Sang Bae, Young-Jun Chung, Jun Xu, Yong Min Kim, Ji-Hun Mo

**Affiliations:** ^1^Department of Otorhinolaryngology-Head and Neck Surgery, Boramae Medical Center, Seoul National University College of Medicine, Seoul, Republic of Korea; ^2^Department of Otorhinolaryngology-Head and Neck Surgery, Chuncheon Sacred Heart Hospital, Hallym University College of Medicine, Chuncheon, Republic of Korea; ^3^Department of Otorhinolaryngology, Dankook University College of Medicine, Cheonan, Republic of Korea; ^4^Beckman Laser Institute Korea, Dankook University College of Medicine, Cheonan, Republic of Korea; ^5^Department of Otorhinolaryngology-Head and Neck Surgery, Research Institute for Medical Science, Chungnam National University School of Medicine, Daejeon, Republic of Korea

## Abstract

**Background and Purpose:**

When house dust mite (HDM), a common allergen, comes into the mucosal membrane, it may stimulate innate immunity. However, the precise role of interleukin- (IL-) 25 in the development of HDM-induced nasal allergic inflammation is still unclear. Therefore, we investigated the role of IL-25 in allergic rhinitis (AR) patients sensitized to HDM.

**Methods:**

To confirm the production of IL-25 in human nasal epithelial cells (HNECs), we stimulated HNECs. IL-25 expression in the nasal mucosa from control, non-AR (NAR) patients, and HDM-sensitized AR patients was assessed using immunohistochemistry, and quantitative reverse transcription PCR. Correlations between IL-25 and other inflammatory markers were explored.

**Results:**

An in vitro study showed significantly elevated concentrations of IL-25 in the HNEC samples with highest doses of HDM. Nasal tissues from AR patients sensitized to HDM showed significantly higher IL-25 expression, compared to those from the control or NAR patients. Moreover, the expression of IL-25 in nasal tissues from AR patients sensitized to HDM was positively associated with Th2 markers, such as ECP and GATA3.

**Conclusions:**

IL-25 expression increased with high-dose HDM stimulation and was related to Th2 markers. Therefore, IL-25 neutralization might offer a new strategy for treating patients with HDM-sensitized AR.

## 1. Introduction

Allergic rhinitis (AR) is a Th2 immune-mediated hypersensitivity in the nasal mucosa characterized by nasal obstruction, rhinorrhea, sneezing, and itching [1]. It is accompanied by an accumulation of eosinophils and mast cells in the nasal mucosa, as well as increased serum levels of antigen-specific IgE [2]. The nasal epithelium, which is the first site of exposure to inhaled antigens, may play an essential role in innate immunity to AR. Recent studies have demonstrated that epithelial cell-derived cytokines, including thymic stromal lymphopoietin (TSLP), interleukin- (IL-) 25, and IL-33, are critical regulators of innate and adaptive immune responses associated with Th2 cytokine-mediated inflammation at nasal mucosal tissues [3–5]. Of these, several studies have described the process by which IL-25 (an IL-17 cytokine family member) can enhance the production of Th2 cell expansion and Th2-type cytokines such as IL-4 and IL-5 [6, 7]. Also, an elevated expression of IL-25 was observed in tissues of patients with asthma, atopic dermatitis, and chronic rhinosinusitis, indicating a possible link between the functions of IL-25 and the exacerbation of allergic disorders [8–11].

House dust mite (HDM; *Dermatophagoides* sp.) is one of the major inhalant allergens that produce patients with perennial AR. Some studies have estimated that 10–20% of the population of any given country is allergic to HDM [12, 13]. As is known, exposure to HDM induces specific antibody production and nasal inflammation by various inflammatory cells, including mast cells, eosinophils, and nasal epithelial cells [14, 15]. Previous animal studies have supported this and described cases of severe nasal symptoms and nasal mucosa remodeling that were observed in the mouse model with HDM-induced AR, but not noted in that with pollen-induced AR [16, 17]. Other animal studies have shown that IL-25 is not crucial for the development of the HDM-induced allergic mice model [18, 19].

To date, regardless of these discrepancies, the role of IL-25 in nasal mucosa of patients with HDM-sensitized AR is still unclear. Therefore, the objective of this study is to investigate the expression of IL-25 in nasal mucosa obtained from patients with HDM-sensitized AR. We also examined the relationship between IL-25 and various inflammatory markers in patients with HDM-sensitized AR.

## 2. Materials and Methods

### 2.1. Subjects

The sinonasal tissue from the inferior turbinate mucosa was obtained through septoplasty from normal control (control; *n* = 8), allergic rhinitis patients (AR; *n* = 14), and nonallergic rhinitis patients (non-AR; *n* = 10). All of the patients provided informed written consents. The internal review board of Dankook University Hospital (number 2012-11-008) approved the study. The exclusion criteria were as follows: (1) the patients were younger than 18 years of age; (2) the patients had prior treatment with antibiotics, systemic or topical corticosteroids, or other immune-modulating drugs during 4 weeks before surgery; and (3) the patients had other chronic sinusitis including rhinosinusitis, antrochoanal polyps, allergic fungal sinusitis, cystic fibrosis, or immotile ciliary disease. Rhinitis was defined as a minimum of 2 nasal complaints (itching, nasal obstruction, rhinorrhea, and sneezing) for more than one year. Patients with a strong positive response to a skin prick test (SPT) were classified as allergic rhinitis patients, and patients with negative SPT response were classified as nonallergic rhinitis patients. A strong positive reaction in the SPT was defined as an allergen to histamine (A/H) ratio of wheel size ≥ 2. The control tissues were obtained from patients without any nasal inflammatory diseases with negative SPT responses during septoplasty.

### 2.2. Immunohistochemistry and Quantitative RT-PCR

Immunohistochemical staining was performed with polink-2, polymerized horseradish peroxidase (HRP), and a broad DAB-Detection System (Golden Bridge International Labs, WA, USA). Briefly, after deparaffinization, the sections were incubated with 3% hydrogen peroxidase to inhibit endogenous peroxidases. Heat-induced epitope retrieval was then performed by microwaving samples in a ten mmol/L citrate buffer (pH 6.0). The sections were incubated for 60 minutes (min) at room temperature in a primary antibody. The primary antibodies were rabbit anti-human IL-25 (1 : 500; Abcam, Cambridge, UK). The sections were incubated in broad-antibody enhancer and polymer-HRP for the rabbit and mouse antibodies. The sections were then stained with the DAB Detection System. Finally, the slides were counterstained with hematoxylin. The numbers of the positive cells in the epithelium, glands, and submucosa were counted in the five densest visual fields (×400) by two independent observers, and the average values were determined. To identify the cellular sources of IL-25, sequential stainings were employed using polymer-HRP and alkaline phosphatase (AP) kits to detect mouse and rabbit primary antibodies for human tissue with Permanent Red and Emerald (Polink DS-MR-Hu C2 Kit; Golden Bridge International Labs). The mouse antimast cell tryptase (1 : 500; Abcam) was mixed with the rabbit anti-human IL-25 (1 : 500; Abcam), applied to the tissue, and then incubated for 30–60 min. The polymer mixtures were made by adding the AP polymer anti-mouse IgG and polymer-HRP anti-rabbit IgG at a 1 : 1 ratio and applied to cover each section. Unless noted otherwise, the manufacturer's instructions were carefully attended to.

In addition, the mRNA expressions of various inflammatory markers in nasal mucosa tissues were determined using quantitative real-time PCR. Total RNA was extracted from the tissue samples by using a TRI reagent (Invitrogen, Carlsbad, CA, USA). One microgram total RNA was reverse transcribed to cDNA using a cDNA synthesis kit (*amfiRivert Platinum* cDNA Synthesis Master Mix, GenDEPOT). Quantitative real-time PCR was carried out using the LightCycler® 480 Probes Master (Roche, Mannheim, Germany). For analysis of IL-25 (Hs03044841_m1), IL-33 (Hs00369211_m1), TSLP (Hs00263639_m1), IFN-*γ* (Hs00989291_m1), and GAPDH (Hs02758991_g1), predeveloped assay reagent kits of primers and probes were purchased from TaqMan Assays (Life Technologies Korea, Seoul, Korea). In addition, a quantitative real-time PCR assay was performed with appropriate primers that specifically amplified T-bet, GATA3, RORC, ECP, and TGF-*β*1. The primers were as follows: T-bet, 5′-GTCAATTCCTTGGGGGAGAT-3′ for the forward primer and 5′-TCATGCTGACTGCTCGAAAC-3′ for the reverse primer; GATA3, 5′-ACCACAACC ACACTCTGGAGGA-3′ for the forward primer and 5′-TCGGTTTCTGGTCTGGATGCCT-3′ for the reverse primer; RORC, 5′-GCTGTGATCTTGCCCAGAACC-3′ for the forward primer and 5′-CTGCCCATCATTGCTGTTAATCC-3′ for the reverse primer; ECP, 5′-TCGGAGTAGATTCCGGGTG-3′ for the forward primer and 5′-GAACCACAGGATACCGTGGAG-3′ for the reverse primer; TGF-*β*1, 5′-TGAACCGGCCTTTCCTGCTTCTCATG-3′ for the forward primer and 5′-GCGGAAGTCAATGTACAGCTGCCGC-3′ for the reverse primer; and GAPDH, 5′-CATGGGTGTGAACCATGAGAA-3′ for the forward primer and 5′-GGTCATGAGTCCTTCCACGAT-3′ for the reverse primer. GAPDH was measured as a housekeeping gene for normalization. Relative gene expression was calculated using the comparative 2^−ΔΔCT^ method.

### 2.3. Cell Culture and Treatments

Healthy adult volunteers were recruited for a nasal brushing of the inferior turbinate to obtain human nasal epithelial cells (HNECs). The samples were placed in a 15 mL conical tube containing 8 mL of DMEM and transported on ice. The samples were then filtered through cell strainers with a pore size of 70 *μ*m and then washed twice with DMEM. After centrifugation, the supernatants were discarded, and the pellets were resuspended in serum-free bronchial epithelial growth medium (BEGM, Lonza Walkersville Inc., Walkersville, MD, USA) supplemented with Single Quots. The cell suspensions were transferred to precoated culture dishes at a concentration of 1 × 10^6^ cells/mL. After incubating them in a tissue culture incubator for 24 hours, the nonadherent cells were removed, and the adherent cells were maintained in BEGM supplemented with Single Quots at 37°C in a humidified atmosphere of 95% air and 5% CO_2_. The culture medium was replaced daily. Subculture was performed when the cells reached 80–90% confluency. Briefly, the culture media was aspirated, and the cells were washed twice with serum-free DMEM. Then, one mL of 0.25% trypsin was added to each dish, and the dishes were incubated at 37°C until the cells became detached. The cells were dislodged by repeatedly pipetting up and down the trypsin solution. The detached cells were then transferred to 15 mL conical tubes with BEGM supplemented with Single Quots. When the cells reached 80–90% confluency, the culture medium was replaced with Single Quots free BEGM for 24 hours to maintain a low basal level of cytokine expression. In this experiment, we used first passaged cells. Before stimulation, the HNECs were cultured in BEBM without hydrocortisone for 24 hours. After 24 hours of starvation, the HNECs were stimulated with *Dermatophagoides farinae* (25, 50, 100, and 200 *μ*g/mL) for 3, 6, 12, 24, and 48 hours.

### 2.4. Statistical Analysis

Statistical analyses were performed with SPSS 18.0 (SPSS Inc., Chicago, Ill). Statistical analyses were performed by using the Kruskal-Wallis and Mann–Whitney *U* tests with a 2-tailed test for unpaired comparisons. The Spearman test was used to determine correlations. The significance level was set at *α* value of 0.05 (^∗^*P* < 0.05, ^∗∗^*P* < 0.010, and ^∗∗∗^*P* < 0.001).

## 3. Results

### 3.1. Induction of IL-25 Expression in Cultured HNECs In Vitro

**Figure 1:**
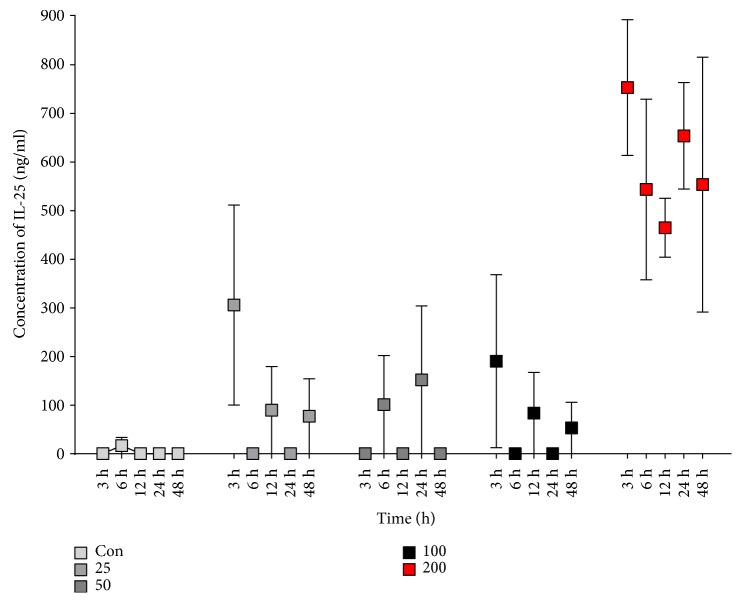
House dust mite-induced IL-25 production in human nasal epithelial cells (HNECs). We used different doses of house dust mite to stimulate the HNECs, including 0, 25, 50, 100, and 200 *μ*g/mL.

To investigate whether HDM induced allergic condition and IL-25 expression in HNECs, we cultured HNECs in air-liquid fashion. We stimulated the HNECs with various concentrations of HDM (25, 50, 100, and 200 *μ*g/mL). In our in vitro study, we found that only the highest concentration of HDM (200 *μ*g/mL) significantly increased IL-25 secretions (Figure 1). The levels of IL-25 in the supernatants of cultured HNECs with 200 *μ*g/mL of HDM were significantly higher than in those with lower concentrations of HDM. This observation suggests that IL-25 levels increase in patients with HDM-sensitized AR.

### 3.2. Expression of Interleukin-25 in Patients with Allergic Rhinitis

**Figure 2:**
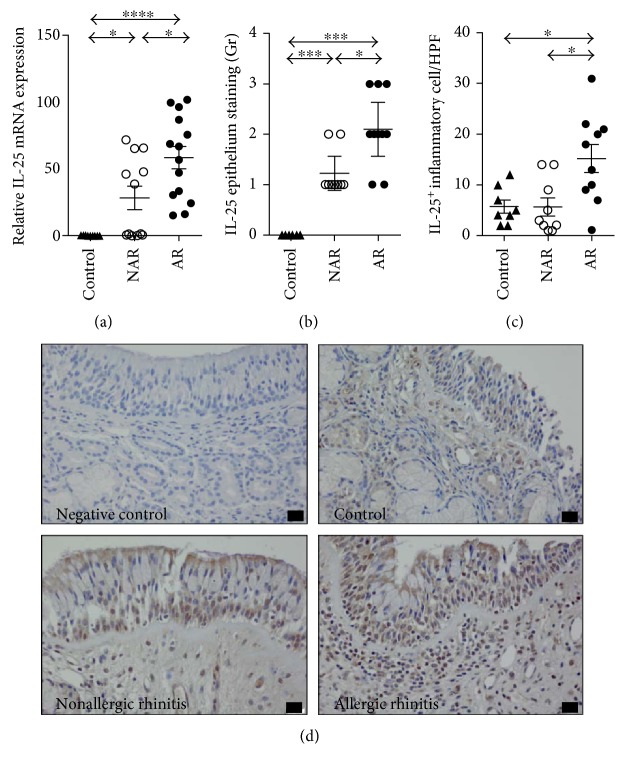
Expression of IL-25 in nasal tissues from control, nonallergic rhinitis patients, and house dust mite-sensitized allergic rhinitis patients. RT-PCR analysis and IHC detection of IL-25 were performed. (a) IL-25 mRNA expression, (b) IL-25 epithelium staining, and (c) IL-25-positive-inflamatory cells in the nasal tissues from each group. (d) Representative images for IL-25 immunohistochemical staining in nasal tissues (^∗^*P* < 0.05, ^∗∗^*P* < 0.010, ^∗∗∗^*P* < 0.001, and ^∗∗∗∗^*P* < 0.0001).

To investigate the expression of IL-25 in the nasal mucosa of patients with HDM-sensitized AR, we performed the real-time quantitative PCR. The expression of IL-25 mRNA was significantly higher in the human nasal mucosa of HDM-sensitized AR compared to that of the control and non-AR patients (Figure 2(a)). Immunohistochemistry (IHC) showed that the expression of IL-25 was higher in the epithelial cells of patients with HDM-sensitized AR than in those of the control subjects and patients with non-AR (Figures 2(b) and 2(d)). In addition, the IL-25-positive inflammatory cells were significantly increased in patients with HDM-sensitized AR, compared to the control subjects and patients with non-AR (Figures 2(c) and 2(d)). Meanwhile, the expression level of IL-33 mRNA was significantly higher in patients with non-AR than in patients with HDM-sensitized AR (Supplementary Fig. 1A available online at https://doi.org/10.1155/2017/3908049). In addition, the expression level of TSLP mRNA was significantly higher in the nasal tissues of HDM-sensitized AR and NAR patients than in those of the control (Supplementary Fig. 1B).

**Figure 3:**
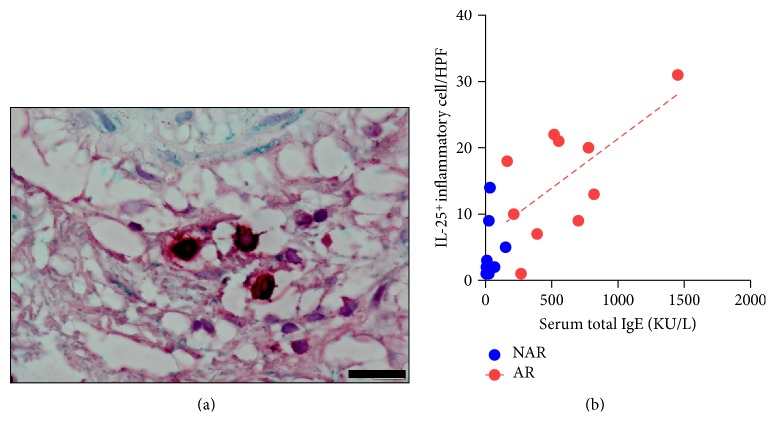
Cellular origin of IL-25 in nasal tissues from house dust mite-sensitized allergic rhinitis: (a) double immunohistochemical staining for IL-25 and mast cells and (b) correlation between the number of IL-25 inflammatory cells and serum total IgE.

Next, we used double IHC staining to identify IL-25 positive cells in the subepithelial layer. Double-positive IL-25 and tryptase cells were frequently detected in patients with HDM-sensitized AR (Figure 3(a)). In addition, we found a meaningful relationship between IL-25-immunoreactive cells and total IgE levels in patients with HDM-sensitized AR (*r* = 0.4169), although there was no such correlation in patients with non-AR (Figure 3(b)).

### 3.3. Correlation between Interleukin-25 mRNA Expression and Inflammatory Markers in Patients with Allergic Rhinitis

**Figure 4:**
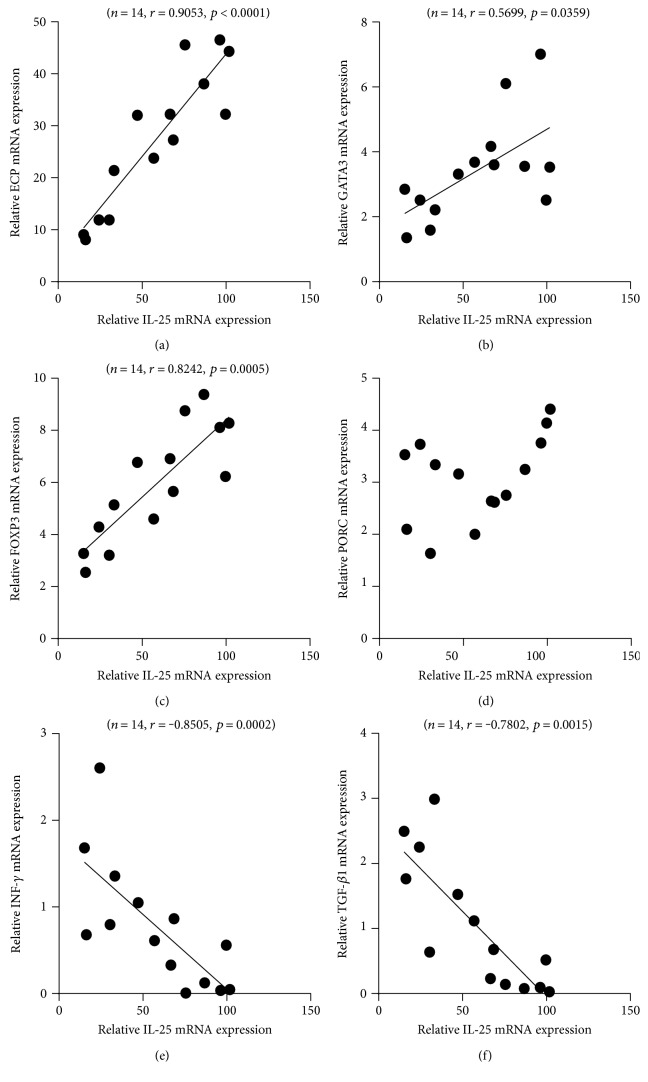
Correlation between IL-25 mRNA expression and inflammatory markers in nasal tissues from house dust mite-sensitized allergic rhinitis: (a) ECP, (b) GATA3, (c) FOXP3, (d) RORC, (e) INF-*γ*, and (f) TGF-*β*1.

To investigate the implication of upregulated IL-25 in patients with HDM-sensitized AR, we examined whether IL-25 expression correlated with other inflammatory markers, such as ECP, GATA3 (a major transcriptional factor in Th2 responses), FOXP3 (a major transcriptional factor in Treg responses), RORC (a major transcriptional factor in Th17 responses), INF-*γ*, and TGF-*β*1. The present study showed that the expression of mRNA for ECP (*r* = 0.9053 and *P* < 0.0001), GATA3 (*r* = 0.5699 and *P* = 0.0359), and FOXP3 (*r* = 0.8242 and *P* = 0.0005) were positively correlated with IL-25 mRNA expression (Figures 4(a), 4(b), and 4(c)), whereas the expression level of IL-25 mRNA bore no correlation with that of RORC (Figure 4(d)). However, IL-25 mRNA expression was negatively associated with INF-*γ* (*r* = −0.8505 and *P* = 0.0002) and TGF-*β*1 (*r* = −0.7802 and *P* = 0.0015) mRNA expression (Figures 4(e) and 4(f)).

## 4. Discussion

The prevalence of AR is increasing, affecting about 18.5% of the Korean population for all ages based on the Korean National Health and Nutrition Survey [20]. In addition, compared with healthy subjects, asthma, nasal polyps, chronic rhinosinusitis, and olfactory dysfunction are more prevalent in patients with AR [21]. Thus, early diagnosis and appropriate treatment of AR are crucial. However, despite a substantial understanding of the clinical characteristics in patients with HDM-sensitized AR, the initial cellular and molecular events that cause susceptible subjects to acquire HDM-induced AR are still unclear.

Recent studies have found the innate immune response to exacerbate inflammation in the nasal airway mucosa [22]. Epithelial cell-derived cytokines, including TSLP, IL-25, and IL-33 produced by airway epithelial cells are important Th2-augmenting cytokines that affect eosinophilic homeostasis and airway inflammation [3–5]. This shows that innate cytokines, such as TSLP, IL-25, and IL-33, are involved in the development of allergic disease by acting as a link between the innate and adaptive airways. HDM allergens could potentially lead to the release of epithelial cell-derived cytokines because sensitization of HDM allergens has resulted in injury to the nasal epithelial cells [23]. Previously, some studies have demonstrated that in allergic reactions, the mugwort pollen allergen was mainly characterized by IgE binding and T-cell proliferation [24, 25], whereas the HDM allergen mediated direct nonspecific damage and allergic reactions in the respiratory epithelium [26, 27]. Specifically, among epithelium-associated cytokines, IL-25 exacerbates allergic inflammation by epithelial cell hyperplasia, mucus secretion, airway hyperresponsiveness, and production of specific Th2 cytokines [28, 29].

To date, several animal studies described that innate cytokines are crucial for the development of acute allergic airway inflammation. However, there were little studies about the relationship between innate cytokines and chronic allergic airway inflammation. To our knowledge, this study is the first to investigate the relationships between IL-25 and chronic allergic airway inflammation, using human nasal tissues. In the present study, we found that in an in vitro assay, HNECs stimulated with high concentrations of HDM produced an increased expression of IL-25. In addition, the expression of IL-25 mRNA level was increased in the nasal mucosa of patients with HDM-sensitized AR, and the number of IL-25-positive-epithelial cells and IL-25-positive-inflammatory cells was significantly higher in the nasal mucosa of patients with HDM-sensitized AR. However, the expression of IL-33 was significantly lower in AR patients than in NAR patients, whereas there was no significant difference in TSLP expression between AR and NAR patients. Consistent with our findings, another study recently demonstrated that IL-25 induced an increased IL-13 expression in the peripheral blood mononuclear cells of HDM-AR patients compared to those of mugwort-AR patients [30]. It means that IL-25 may play a more important role in chronic allergic airway inflammation, such as AR induced by HDM than TSP or IL-33. Therefore, to support our conclusion, we need to investigate the role of IL-25 in the development of HDM-induced human allergic nasal inflammation further.

Interestingly, in the analysis of the IL-25 and inflammatory markers, we observed a meaningful relationship between the IL-25-positive inflammatory cells and total IgE. Moreover, the IL-25 mRNA level was significantly correlated with inflammatory markers such as ECP and GATA3 for the Th2 immune response. These findings suggest that IL-25 may play a major role as one of the mediators for the development of Th2 immune response in patients with HDM-induced AR. Although other prior studies on the HDM-induced allergic mice model have demonstrated that IL-25 is unnecessary for Th2 priming and the subsequent effector responses to the HDM allergens [18, 19], we believe this discrepancy can be explained by their reliance on acute allergic mice models, as opposed to our study's use of human nasal tissues—a type of chronic model for HDM-induced AR. Therefore, to more elucidate the role of IL-25 in AR, we need further studies such as IL-25 blocking antibody or IL-25 knockout mice studies, using animal models for chronic allergic airway inflammation.

## 5. Conclusions

In the present study, we have confirmed the increased production of IL-25 in cultured HNECs, when stimulated with high concentrations of HDM. Particularly interesting are the increased expression of IL-25 in nasal tissues from patients sensitized with HDM-induced AR and the positive correlation between the IL-25 and Th2 markers observed in the present study. These findings suggest that IL-25 may involve in the development of Th2 immune response in HDM-induced AR. Therefore, IL-25 neutralization might be a potential approach for the treatment of patients with HDM-sensitized AR.

## Supplementary Material

Supplementary Fig. 1. The expression of IL-33 and TSLP mRNA in nasal tissues from control, non-allergic rhinitis, and house dust mite sensitized allergic rhinitis.

## References

[1] Bousquet J., Khaltaev N., Cruz A. A. (2008). Allergic Rhinitis and its Impact on Asthma (ARIA) 2008 update (in collaboration with the World Health Organization, GA(2)LEN and AllerGen). *Allergy*.

[2] Skoner D. P. (2001). Allergic rhinitis: definition, epidemiology, pathophysiology, detection, and diagnosis. *The Journal of Allergy and Clinical Immunology*.

[3] Saenz S. A., Taylor B. C., Artis D. (2008). Welcome to the neighborhood: epithelial cell-derived cytokines license innate and adaptive immune responses at mucosal sites. *Immunological Reviews*.

[4] Kouzaki H., Matsumoto K., Kato T., Tojima I., Shimizu S., Shimizu T. (2016). Epithelial cell-derived cytokines contribute to the pathophysiology of eosinophilic chronic rhinosinusitis. *Journal of Interferon & Cytokine Research*.

[5] Mjosberg J. M., Trifari S., Crellin N. K. (2011). Human IL-25- and IL-33-responsive type 2 innate lymphoid cells are defined by expression of CRTH2 and CD161. *Nature Immunology*.

[6] Morita H., Arae K., Unno H. (2015). IL-25 and IL-33 contribute to development of eosinophilic airway inflammation in epicutaneously antigen-sensitized mice. *PLoS One*.

[7] Iwakura Y., Ishigame H., Saijo S., Nakae S. (2011). Functional specialization of interleukin-17 family members. *Immunity*.

[8] Corrigan C. J., Wang W., Meng Q. (2011). Allergen-induced expression of IL-25 and IL-25 receptor in atopic asthmatic airways and late-phase cutaneous responses. *The Journal of Allergy and Clinical Immunology*.

[9] Wang Y. H., Angkasekwinai P., Lu N. (2007). IL-25 augments type 2 immune responses by enhancing the expansion and functions of TSLP-DC-activated Th2 memory cells. *The Journal of Experimental Medicine*.

[10] Hvid M., Vestergaard C., Kemp K., Christensen G. B., Deleuran B., Deleuran M. (2001). IL-25 in atopic dermatitis: a possible link between inflammation and skin barrier dysfunction?. *The Journal of Investigative Dermatology*.

[11] Shin H. W., Kim D. K., Park M. H. (2015). IL-25 as a novel therapeutic target in nasal polyps of patients with chronic rhinosinusitis. *The Journal of Allergy and Clinical Immunology*.

[12] Sunyer J., Jarvis D., Pekkanen J. (2004). Geographic variations in the effect of atopy on asthma in the European Community Respiratory Health Study. *The Journal of Allergy and Clinical Immunology*.

[13] Arshad S. H., Tariq S. M., Matthews S., Hakim E. (2001). Sensitization to common allergens and its association with allergic disorders at age 4 years: a whole population birth cohort study. *Pediatrics*.

[14] Rydell-Tormanen K., Johnson J. R., Fattouh R., Jordana M., Erjefalt J. S. (2008). Induction of vascular remodeling in the lung by chronic house dust mite exposure. *American Journal of Respiratory Cell and Molecular Biology*.

[15] Shin S. Y., Choi S. J., Hur G. Y. (2009). Local production of total IgE and specific antibodies to the house dust mite in adenoid tissue. *Pediatric Allergy and Immunology*.

[16] Ciprandi G., Cirillo I., Pistorio A., La Grutta  S. (2008). Relationship between rhinitis duration and worsening of nasal function. *Otolaryngology--Head and Neck Surgery*.

[17] Ogita-Nakanishi H., Nabe T., Mizutani N., Fujii M., Kohno S. (2009). Absence of nasal blockage in a Japanese cedar pollen-induced allergic rhinitis model mouse. *Allergology International*.

[18] Nakanishi W., Yamaguchi S., Matsuda A. (2013). IL-33, but not IL-25, is crucial for the development of house dust mite antigen-induced allergic rhinitis. *PLoS One*.

[19] Chu D. K., Llop-Guevara A., Walker T. D. (2013). IL-33, but not thymic stromal lymphopoietin or IL-25, is central to mite and peanut allergic sensitization. *The Journal of Allergy and Clinical Immunology*.

[20] Ahn J. C., Kim J. W., Lee C. H., Rhee C. S. (2016). Prevalence and risk factors of chronic rhinosinusitus, allergic rhinitis, and nasal septal deviation: results of the Korean National Health and Nutrition Survey 2008-2012. *JAMA Otolaryngology - Head & Neck Surgery*.

[21] Rhee C. S., Wee J. H., Ahn J. C. (2014). Prevalence, risk factors and comorbidities of allergic rhinitis in South Korea: The Fifth Korea National Health and Nutrition Examination Survey. *American Journal of Rhinology & Allergy*.

[22] Kim H. Y., DeKruyff R. H., Umetsu D. T. (2010). The many paths to asthma: phenotype shaped by innate and adaptive immunity. *Nature Immunology*.

[23] Wang J. Y. (2013). The innate immune response in house dust mite-induced allergic inflammation. *Allergy, Asthma & Immunology Research*.

[24] Himly M., Jahn-Schmid B., Dedic A. (2003). Art v 1, the major allergen of mugwort pollen, is a modular glycoprotein with a defensin-like and a hydroxyproline-rich domain. *FASEB Journal*.

[25] Jahn-Schmid B., Kelemen P., Himly M. (2002). The T cell response to art v 1, the major mugwort pollen allergen, is dominated by one epitope. *Journal of Immunology*.

[26] Wan H., Winton H. L., Soeller C. (1999). Der p 1 facilitates transepithelial allergen delivery by disruption of tight junctions. *The Journal of Clinical Investigation*.

[27] Herbert C. A., King C. M., Ring P. C. (1995). Augmentation of permeability in the bronchial epithelium by the house dust mite allergen der p1. *American Journal of Respiratory Cell and Molecular Biology*.

[28] Fort M. M., Cheung J., Yen D. (2001). IL-25 induces IL-4, IL-5, and IL-13 and Th2-associated pathologies in vivo. *Immunity*.

[29] Hurst S. D., Muchamuel T., Gorman D. M. (2002). New IL-17 family members promote Th1 or Th2 responses in the lung: in vivo function of the novel cytokine IL-25. *Journal of Immunology*.

[30] Fan D., Wang X., Wang M. (2016). Allergen-dependent differences in ILC2s frequencies in patients with allergic rhinitis. *Allergy, Asthma & Immunology Research*.

