# Supplementary material for: IL-25 Could Be Involved in the Development of Allergic Rhinitis Sensitized to House Dust Mite
Source: Mediators Inflamm. 2017 Aug 23;2017:3908049. doi: 10.1155/2017/3908049 (PMC5587973; doi:10.1155/2017/3908049)

Supplementary Fig. 1. The expression of IL-33 and TSLP mRNA in nasal tissues from control, non-allergic rhinitis, and house dust mite sensitized allergic rhinitis.

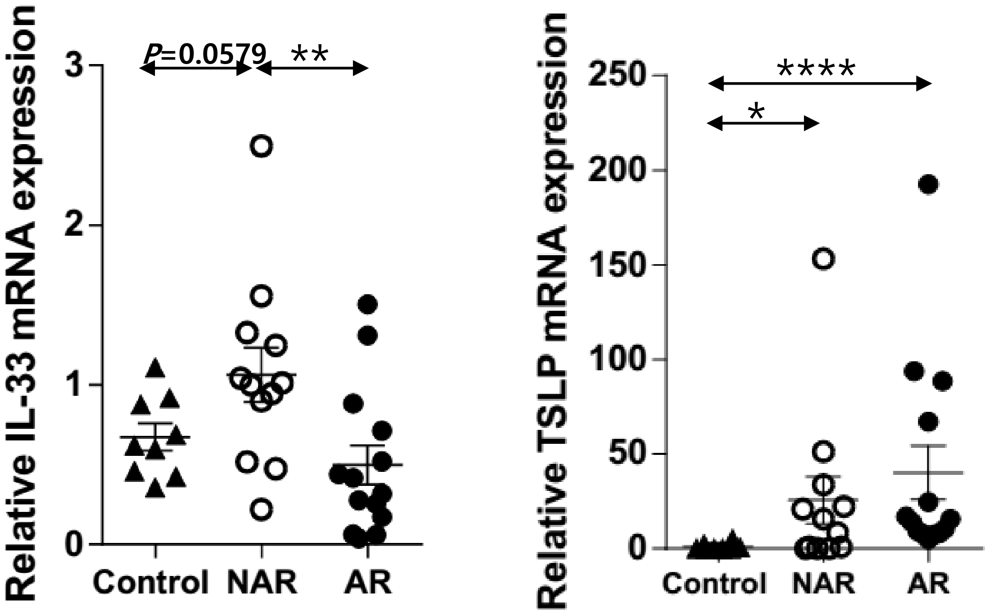

Supplement: Supplementary file 1 — Supplementary Fig. 1. The expression of IL-33 and TSLP mRNA in nasal tissues from control, non-allergic rhinitis, and house dust mite sensitized allergic rhinitis. [file 3908049.f1.pdf]
